# The Influence of Feedback on Task-Switching Performance: A Drift Diffusion Modeling Account

**DOI:** 10.3389/fnint.2018.00001

**Published:** 2018-02-02

**Authors:** Russell Cohen Hoffing, Povilas Karvelis, Samuel Rupprechter, Peggy Seriès, Aaron R. Seitz

**Affiliations:** ^1^UCR Brain Game Center, Department of Cognitive Psychology, University of California, Riverside, Riverside, CA, United States; ^2^School of Informatics, University of Edinburgh, Edinburgh, United Kingdom

**Keywords:** task-switching, feedback, drift diffusion model, learning, executive function, training

## Abstract

Task-switching is an important cognitive skill that facilitates our ability to choose appropriate behavior in a varied and changing environment. Task-switching training studies have sought to improve this ability by practicing switching between multiple tasks. However, an efficacious training paradigm has been difficult to develop in part due to findings that small differences in task parameters influence switching behavior in a non-trivial manner. Here, for the first time we employ the Drift Diffusion Model (DDM) to understand the influence of feedback on task-switching and investigate how drift diffusion parameters change over the course of task switch training. We trained 316 participants on a simple task where they alternated sorting stimuli by color or by shape. Feedback differed in six different ways between subjects groups, ranging from No Feedback (NFB) to a variety of manipulations addressing trial-wise vs. Block Feedback (BFB), rewards vs. punishments, payment bonuses and different payouts depending upon the trial type (switch/non-switch). While overall performance was found to be affected by feedback, no effect of feedback was found on task-switching learning. Drift Diffusion Modeling revealed that the reductions in reaction time (RT) switch cost over the course of training were driven by a continually decreasing decision boundary. Furthermore, feedback effects on RT switch cost were also driven by differences in decision boundary, but not in drift rate. These results reveal that participants systematically modified their task-switching performance without yielding an overall gain in performance.

## Introduction

Task-switching is an important cognitive skill that facilitates our ability to choose appropriate behavior in a varied and changing environment. Task-switching ability changes throughout the lifespan (Kray and Lindenberger, 2000; Cepeda et al., 2001; Davidson et al., 2006; Huizinga et al., 2006; Wasylyshyn et al., 2011), suggesting that this ability may be malleable. Consistent with this, training studies show that task-switching can, at least in certain circumstances, be improved through training (Minear and Shah, 2008; Karbach and Kray, 2009; Strobach et al., 2012). These training paradigms are promising as a method to improve task-switching functions but give rise to inconsistent learning outcomes (Minear and Shah, 2008; Karbach and Kray, 2009; Pereg et al., 2013). It is likely that part of these training outcome inconsistencies are due to the use of different task structures and parameters across studies (Vandierendonck et al., 2010). In task-switching training, different preparatory times (Monsell, 2003), cues (Monsell, 2003) and predictability of the task switch (Minear and Shah, 2008) have been found to influence performance and learning. In the present article, we add to this literature by examining the influence of feedback on training, which has not been well explored in the context of task-switching.

Feedback on the accuracy and timeliness of one’s performance can provide critical information to guide behavior (Yeung et al., 2004). While the role of external feedback is critical to achieve accurate proficiency in tasks where the correct response can only be learned operantly (such as in the Wisconsin Card Sorting Task), feedback can be less important in tasks where the participant knows which answers are correct and those which are not (Herzog and Fahle, 1997; Seitz et al., 2006; Liu et al., 2014). For example, in typical task-switching tasks, participants will know whether their responses are correct or incorrect and thus feedback may be more relevant as a motivational signal rewarding participants for a job well done (Seitz and Dinse, 2007; Seitz et al., 2009). For example, feedback has been used to study motivated decision making by associating different reward values to correct stimulus-response mappings with results suggesting that higher valued responses are related to increases in performance (Botvinick and Braver, 2015). Consistent with this motivational framework, in some cases people show more learning when falsely inflated feedback is provided than when accurate feedback is provided, suggesting models where feedback serves to increase learning rates rather than to supervise learning (Shibata et al., 2009). On the other hand, feedback meant to provide motivation can also impair learning (Katz et al., 2014), perhaps due to the distracting role that some feedback can have during task performance. Given these conflicting roles of feedback in the literature, we sought to determine both the extent to which feedback alters performance during task-switching and to understand what components of the decision process are altered.

While multiple studies have looked at which task parameters influence task-switching learning and performance, few have shed light on the changes to decision processes that underlie that learning. With current computational techniques it is possible to model decision processes during task-switching. In particular, the Drift Diffusion Model (DDM; Ratcliff, 1978) decomposes the decision process into different components, addressing biases, information integration rates, and the amount of accumulated information required to make a decision; each component offers insight into changes in the decision process responsible for differences at the behavioral level. A benefit of the DDM is that it can jointly account for both the reaction time (RT) and accuracy distributions providing a more informative description of behavior than summary statistics such as the mean RT. The DDM has been successfully applied to understanding processes involved in a variety of two-alternative forced choice tasks, such as recognition memory tasks, lexical decision and visual-scanning tasks (Ratcliff, 1978; Strayer and Kramer, 1994; Ratcliff and Rouder, 1998; Ratcliff and McKoon, 2008). Previous studies that applied the DDM to understand task-switching (Karayanidis et al., 2009; Madden et al., 2009; Schmitz and Voss, 2012), have found that participants modify decision processes on a trial-by-trial basis. In particular, Schmitz and Voss (2012) found that drift rates were higher for non-switch trials than switch trials and interpreted this to reflect interference from the previous trial. Furthermore, results indicated that decision boundaries were higher for switch trials than non-switch trials, which was interpreted to reflect increased caution on switch trials.

Here, for the first time we employ the DDM to understand the influence of feedback on task-switching and how drift diffusion parameters change over the course of task switch training. To accomplish this, we trained 316 participants on a simple task-switching task where they alternated sorting stimuli by color or by shape. Feedback differed in six different ways between subjects ranging from No Feedback (NFB) to a variety of manipulations addressing trial-wise vs. Block Feedback (BFB), rewards vs. punishments, payment bonuses and different payouts depending upon hard or easy trial types. This way we could look at how different feedback conditions may lead to different patterns of performance change across 10 blocks of training trials. Results showed that the most significant distinction was between the NFB condition compared to the other feedback conditions, and that while RT and accuracy data provided a pattern of results that was difficult to interpret, the DDM model parsimoniously accounted for the data through differences in both integration rate and decision boundaries.

## Materials and Methods

### Participants

A total of 316 participants (Female = 202; Age: Mean = 19.66 years, STD = 2.84 years) were recruited to take part in the study. All participants had normal or corrected-to-normal visual acuity and received course credit for the 1 h session. This study was carried out in accordance with the approval of the University of California, Riverside Human Research Review Board. All subjects gave written informed consent in accordance with the Declaration of Helsinki. The protocol was approved by the University of California, Riverside Human Research Review Board.

### General Procedure and Training Task

**Figure 1:**
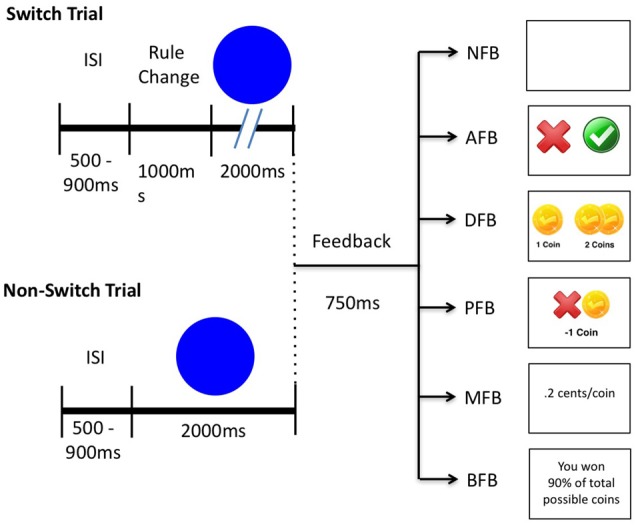
Schematic depicting switch trials, non-switch trials and feedback conditions. A blank screen is presented for an inter-stimulus-interval (ISI) of 500–900 ms. In switch trials participants are cued to a rule change for 1000 ms while in non-switch trials no cue is presented. Afterwards a stimulus appears for 2000 ms or until a response after which feedback is presented for 750 ms according to the following conditions: in No Feedback (NFB) a blank screen; in Accuracy Feedback (AFB) a green check for correct responses and a red “x” for incorrect responses; in Difficulty Feedback (DFB) one coin for a correct response and a bonus of either one or three coins if a fast response was made and a red “x” for incorrect responses; in Punishment Feedback (PFB), the same bonuses as in the DFB but also a minus one coin for incorrect responses; in Monetary Feedback (MFB) the same feedback as PFB but each coin was worth 0.2 cents; in Block Feedback (BFB) the same feedback as PFB but also overall accuracy after each block.

Participants trained for one session on a task-switching task. An Apple Mac Mini running MATLAB (Mathworks, Natick, MA, USA) and Psychtoolbox Version 3.0.8 was used to generate stimuli (Brainard, 1997; Pelli, 1997). Each session is comprised of 10 training blocks and four pre/post blocks (2 pre, 2 post) with 60 trials a block for a total of 840 trials. In the main task, participants switched between two tasks categorizing colored shapes (Figure 1). In Task 1 participants categorized images by color (Blue or Green) and in Task 2 stimuli are categorized by shape (Circle or Square). In the first and last pre/post block, novel stimuli and tasks (i.e., Tigers and Lions, Sitting and Standing) were used to test transfer to untrained stimuli. No feedback was presented in any of the pre/post blocks. Eight stimuli were randomly chosen from a set of 25 stimuli comprised of multiple exemplars of the rule categories. For example, five shades of Blue and Green, and five sizes of Circles and Squares were used. A relatively large set of stimuli was chosen because previous research suggests that increased stimulus variability facilitates transfer (Wang et al., 2014; Deveau et al., 2015). Trials in which a switch occurs are referred to as “switch trials” and trials in which a task repeats are referred to as “non-switch trials”. In both trial types stimuli appeared for 2 s or until a response was made after which a blank screen was displayed for a randomized inter-trial-interval (ITI) of 0.5–0.9 s. Switch trials occurred every four trials and a cue was displayed for 1 s before stimulus presentation (i.e., “Rule Change” was displayed) whereas on non-switch trials stimuli were presented immediately.

### Experimental Manipulation on Feedback

Participants were randomly assigned to one of the six training conditions based on subject number (see Figure 1). Conditions consisted of NFB (*N* = 51), Accuracy Feedback (AFB, *N* = 53), Difficulty Aware (DFB, *N* = 57), Punishment Feedback (PFB), *N* = 55), Monetary Bonus (MFB, *N* = 52), or BFB, *N* = 48). These conditions reflect standard manipulations of feedback seen across the literature, but their influence on task switching performance and training have not been systematically tested. Each condition only differed on the 10 training blocks. Feedback (if provided) was given in the form of gold coins immediately after a response and displayed for 750 ms. Standard correct responses received one gold coin, and bonuses were provided based on difficulty and speed in relation to a 600 ms response time criterion. The speed criterion was taken from the average RT (600 ms) from a pilot study of 306 participants.

In the NFB condition, which served as our control condition, participants did not receive any feedback and instead viewed a blank screen for 750 ms. In the AFB condition participants were only given feedback indicating correct or incorrect responses to assess how simple motivational signals influence performance. In the DFB condition, participants received bonuses according to performance during difficult trials as described in the bonus structure above to assess the influence of specific motivational information. In the DFB condition, we took into account the fact that responses are slower on switch trials by giving one bonus coin if an accurate response is within 20% of the speed criterion on switch trials and 5% of the speed criterion on non-switch trials, and three bonus coins if an accurate response is within 5% of the speed criterion on switch trials. In the PFB participants received feedback as described above, however incorrect or slow responses were punished with a −1 gold coin to assess the effect of loss aversion. The MFB condition was the same as the PFB condition except that participants received 0.2 cents per coin they won to assess the influence of monetary incentives. The BFB condition was the same as the PFB condition except participants received feedback at the end of each block indicating the percent of total coins received to assess how block-wise information impacts performance.

### Data Analysis

Out of 316 participants, 11 were excluded based on a 80% accuracy criterion, which corresponds to about two standard deviations from the mean (see Supplementary Figure S1 for distributions). In addition to analyzing mean RT and accuracy across participants, we looked at switch cost which is defined as a ratio of the RTs on switch and non-switch trials to determine relative changes in performance. Defining switch cost as a ratio (as opposed to the difference) better accounts for relative changes from baseline RT (e.g., a 200 ms slow down represents a greater change from a 400 ms baseline than from a 1200 ms baseline). Furthermore, this allows for simpler comparison between switch costs as estimated from RT and estimated from model parameters. We note that using switch cost differences rather than switch cost ratios produced qualitatively similar results (see Supplementary Figure S2). Finally, an alpha level of 0.05 was used for all statistical tests.

### Modeling

**Figure 2:**
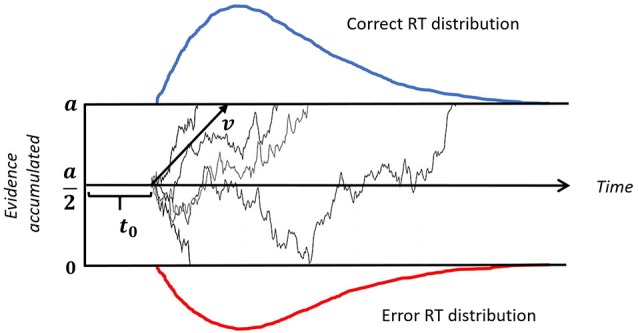
Illustration of a drift-diffusion model. Thin black lines represent trajectories of individual random walks. Each walk captures noisy accumulation of evidence in time on a single trial. The speed of accumulation is determined by the drift-rate (v). A response is initiated when either of the boundaries (a or 0) is reached. The upper (blue) and lower (red) panels represent reaction time (RT) distributions for correct and incorrect responses, respectively. The time gap between the onset of a stimulus and start of the evidence accumulation is non-decision time, denoted by t_0_.

To better understand how the different feedback conditions influence decision processes, we fitted a DDM (see Figure 2) to the data. DDM construes the decision making process as a random walk which can be simulated using the equation:

(1) W(t+dt)=W(t)+v·dt+n,

where dt is a time step in simulation, v is the mean drift rate and n is random Gaussian noise. W is a location at any given time between the two boundaries 0 and *a*. The decision is made once either of the boundaries is reached. In our case, reaching 0 corresponds to an incorrect response, while reaching *a* corresponds to a correct response. W(*t* = 0) is a starting point that reflects any bias towards a particular stimulus, but since we fit correct/incorrect responses across all stimuli no such bias is possible, therefore we fixed the starting point at an equal distance from the two boundaries, that is W(*t* = 0) = *a*/2.

*Drift rate (v)* reflects the efficiency with which stimulus information is used to select a response; it can be affected by task difficulty, individual differences in intelligence and working memory capacity, as well as motivation, fatigue or inattention (Schmiedek et al., 2007). In the task-switching paradigm, the drift rate might be affected by the activation of stimulus-response mapping rules task-set biasing, or other factors contributing to task readiness (Schmitz and Voss, 2012).

*Decision Boundary (a)* is normally regarded as a measure of caution or conservatism: larger values of the boundary result in slower responses but higher accuracy (Schmiedek et al., 2007). In other words, it captures speed-accuracy trade-off effects. Some studies suggest that in a task-switching paradigm, the decision threshold can vary on trial-by-trial basis: caution can be reduced for predictable repeat trials (Schmitz and Voss, 2012) or increased for predictable switch trials (Karayanidis et al., 2009).

*Non-decision time (t_0_)* is thought to reflect the duration of pre-decision processes such as encoding, preparation of the right task set, and motor processes of the response system (Ratcliff and McKoon, 2008). Previous studies have found that, non-decision time on switch trials was the same as on non-switch trials with a cue-stimulus interval as low as 600 ms (Madden et al., 2009). Because we used 1500–1900 ms cue-stimulus interval, we assumed the non-decision time to be fixed across switch and non-switch trials.

To fit the DDM we used a hierarchical Bayesian parameter estimation toolbox (Wiecki et al., 2013). This enabled us to get robust fits as it makes use of commonalities among individuals (both individual and group-level parameters are fitted at once, where group-level parameters function as a prior for individual fits). This is especially advantageous in data sets with small number of trials. DDM parameters can be very sensitive to outliers in individual responses, especially when arbitrarily quick responses are made. To account for the fraction of random responses, we assumed a lapse rate of 10% (i.e., drawn from a uniform distribution). The precise value of the assumed lapse rate, as long as it is not too low, does not have much influence on the estimated model parameters; values in the range of 1%–10% have been shown to work well in DDM (Wiecki et al., 2013).

## Results

### Behavioral Data

**Figure 3:**
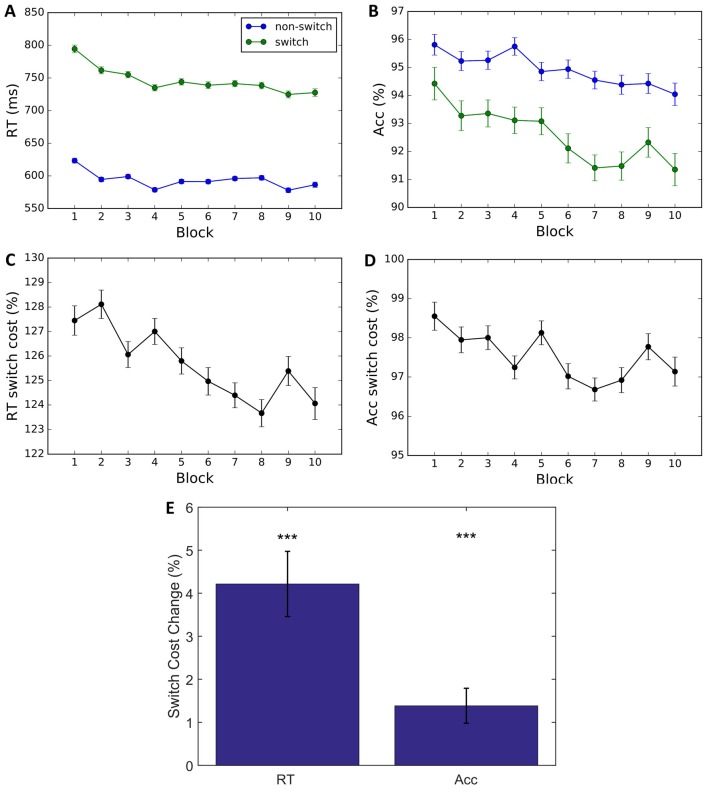
Behavioral data. **(A,B)** Average RT and percent correct by block. Results indicate a decrease in Average RT and Accuracy for switch and non-switch trials. **(C,D)** Switch cost is calculated by dividing switch by non-switch performance. A larger decrease in switch trials is reflected in a reduction in switch cost RT and switch cost accuracy. **(E)** Switch cost change is calculated by subtracting Block 10 performance from Block 1. The bar plots indicate that change in RT and accuracy switch costs are significantly greater than 0. Error bars represent within-subject errors. ***p < 0.001.

To understand how the feedback manipulations influenced task performance, we performed a mixed analysis of variance (ANOVA) on Block × Trial Type × Feedback Condition with subjects as random effects. Results indicate a significant main effect of Block (*F*_(9,295)_ = 16.87, *p* < 0.001, $\eta_{\text{p}}^{2}$ = 0.007) and interaction for Block × Trial Type for RT (*F*_(9,295)_ = 5.61, *p* < 0.001, $\eta_{\text{p}}^{2}$ = 0.144) but not accuracy (*F*_(9,295)_ = 1.68, *p* = 0.088) suggesting decreases in RT switch cost. But this interpretation is complicated due to a main effect of Block on Accuracy (*F*_(9,295)_ = 7.94, *p* < 0.001, $\eta_{\text{p}}^{2}$ = 0.195), indicating a significant decrease in accuracy over training (Block 1: 95.07%, Block 10: 92.64%, Figures 3A,B). This result suggests that a decrease in RT switch cost is partly due to a speed-accuracy trade-off (Figures 3C,D). To quantify changes in switch cost over time, we performed paired *t*-tests on changes in switch cost between Blocks 1 and 10, and found a significant decrease in both RT and accuracy (Figure 3E; *t*_(304)_ = 988.1, *p* < 0.001, *d* = 65.377; and *t*_(304)_ = 606.3, *p* < 0.001, *d* = 80.397, respectively), with a proportionately greater change in RT than in accuracy, suggesting a reduction in switch costs. Altogether, direct examination of RT and accuracy provide a mixed story: it is unclear whether something other than a speed-accuracy trade-off, such as learning, is occurring.

**Figure 4:**
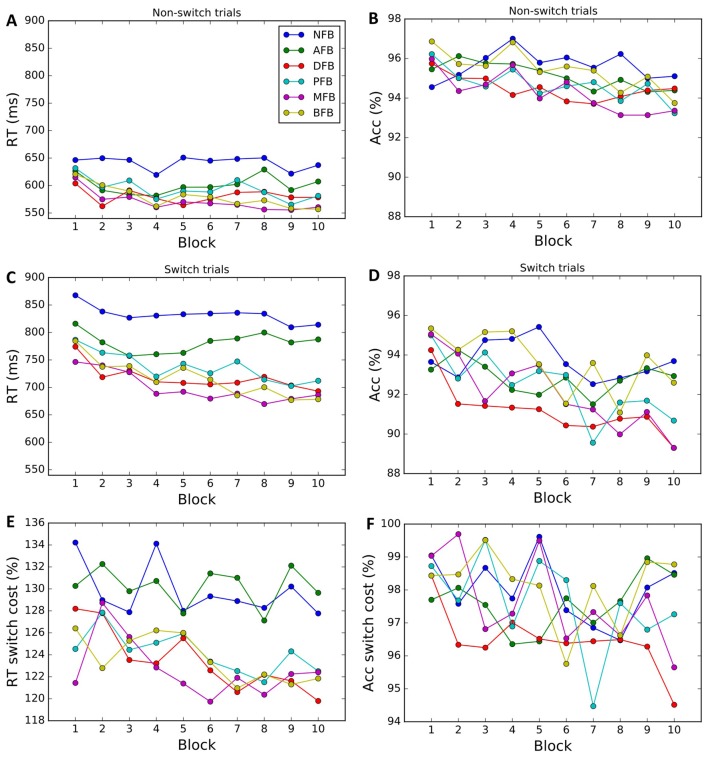
Behavioral data by condition. Average RTs and accuracy for non-switch **(A,B)** and switch trials **(C,D)** in each block and corresponding switch costs **(E,F)**. Each color corresponds to a different condition (NFB, No feedback; AFB, Accuracy feedback; correct or incorrect feedback, DFB, Difficulty aware feedback; bonus if fast and correct, PFB, Punishment feedback; punishment, −1 coin for incorrect responses, MFB, Monetary feedback; same as PFB, but each coin is worth 0.2 cents, BFB, Block feedback; same as PFB, but at the end of each block they are given block accuracy performance).

**Figure 5:**
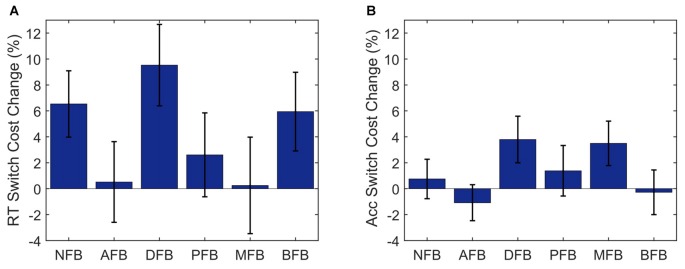
Switch cost by condition. Change in Switch Cost from blocks 1 to 10 for RT **(A)** and Accuracy **(B)** by Condition. NFB, No Feedback; AFB, Accuracy Feedback; DFB, Difficulty Aware Feedback; MFB, Monetary Feedback; BFB, Block Feedback. Error bars represent standard errors.

We next examined whether the different feedback conditions impacted performance and learning (see Figures 4A–F). Results indicate a main effect of Condition (*F*_(5,299)_ = 3.868, *p* = 0.002, $\eta_{\text{p}}^{2}$ = 0.061) on RT. The two-way interaction between Condition × Block found for RT (*F*_(45,1475)_ = 1.67, *p* = 0.004, $\eta_{\text{p}}^{2}$=0.235) but not for accuracy (*F*_(45,1475)_ = 1.03, *p* = 0.425), suggests that task feedback also had an effect on learning, where with time participants became faster in some of the feedback conditions. To investigate which conditions are driving the interaction, we conducted *post hoc* two-tailed paired *t*-tests comparing the average RT on block 1 and 10 and found that DFB (*t*_(54)_ = 2.488, *p* = 0.016, *d* = 0.595), PFB (*t*_(51)_ = 3.084, *p* = 0.003, *d* = 0.611), MFB (*t*_(54)_ = 2.488, *p* = 0.003, *d* = 0.615), and BFB (*t*_(54)_ = 2.488, *p* < 0.001, *d* = 0.595) showed a significant differences whereas NFB (*t*_(54)_ = 2.488, *p* = 0.222) and AFB (*t*_(54)_ = 2.488, *p* = 0.124) did not. These results suggest that feedback conditions that convey information in relation to switch performance are driving the Condition × Block interaction. The three-way interaction term between Condition × Trial-Type × Block, however, failed to reach significance for either RT (*F*_(45,1475)_ = 1.0, *p* = 0.458) or accuracy (*F*_(45,1475)_ = 0.8, *p* = 0.854), suggesting that different feedback conditions had minimal effect on the change in task switching performance over training. To look at changes in switch cost over the course of training by condition we conducted a one-way ANOVA (Figure 5) on the change in switch cost between Block 1 and 10 and failed to find a significant difference across conditions in either RT (*F*_(5,299)_ = 1.41, *p* = 0.222) or Accuracy (*F*_(5,299)_ = 1.39, *p* = 0.229). These results suggest that while feedback affected overall task performance and learning, it did not significantly impact changes in switch costs.

### Modeling

We used a DDM to investigate what aspects of the decision process are affected by training and feedback and to determine to what extent speed-accuracy trade-off was driving the observed behavioral effects. We fitted a set of DDMs, each of which differed in what parameters were allowed to vary across blocks and trial types. If conditioning a parameter on trial type or block improves the model fit, it means that that parameter does vary across trial types or blocks, respectively. The set of models were compared based on Deviance Information Criterion (DIC), which is a standard measure for comparing hierarchical models (Wiecki et al., 2013). In the following, we present only the results for our winning model, which conditions drift rate (v) and decision boundary (a) on trial type and block (see Supplement Table S1 for the alternative models).

**Figure 6:**
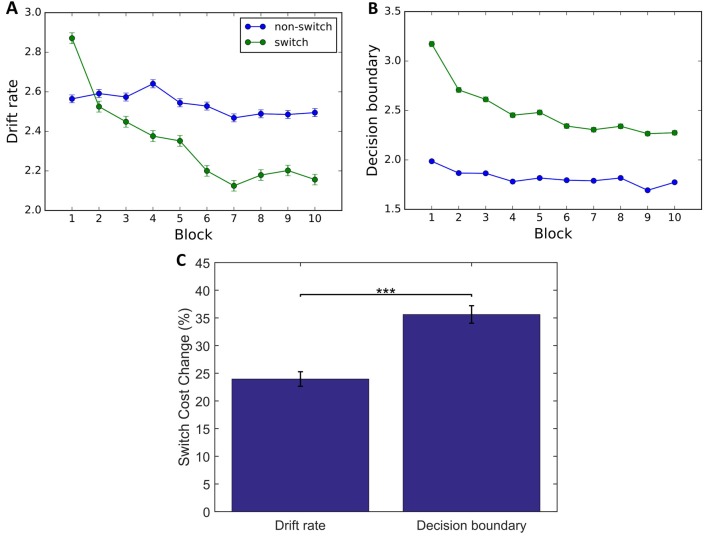
Drift diffusion model (DDM) data. Group level parameters for all participants (*n* = 305) for switch trials (green) and non-switch trials (blue). **(A,B)** Results indicate a decrease in drift rate **(A)** and decision boundary **(B)**. **(C)** A larger change in decision boundary than in drift rate from blocks 1 to 10 indicates that the decrease in RT and Accuracy is driven by a decrease in decision boundary. Error bars represent within-subject errors. ***p < 0.001.

First, we looked at the change in parameters on switch and non-switch trials averaged across conditions (Figures 6A,B). As with the behavioral data, we performed a 3-way mixed ANOVA to determine changes in parameters driving overall performance and switch cost effects. We found that there was a significant main effect of Block on drift rate (*F*_(9,295)_ = 96.17, *p* < 0.001, $\eta_{\text{p}}^{2}$ = 0.619) and decision boundary (*F*_(9,295)_ = 82.03, *p* < 0.01, $\eta_{\text{p}}^{2}$ = 0.714). For the drift rate this decrease was significantly different between trial types (Block × Trial Type (*F*_(45,1475)_ = 54.14, *p* < 0.001, $\eta_{\text{p}}^{2}$ = 0.663) with a greater decrease in switch trials (Block 1: 2.87; Block 10: 2.15) than in non-switch trials (Block 1: 2.58; Block 10: 2.49). The same was true for the decision boundary (Block × Trial type: *F*_(45,1475)_ = 62.80, *p* < 0.001, $\eta_{\text{p}}^{2}$ = 0.613), with a greater decrease in switch (Block 1: 3.22; Block 10: 2.45) than in non-switch trials (Block 1: 2.00; Block 10: 1.75). While a decrease in drift rate alone would result in increased RT and decreased accuracy, a decrease in decision boundary would lead to decreased RT and also decreased accuracy. Taking this into consideration, the results suggest that the observed decrease in RT switch cost over the course of training was solely due to the decrease in decision boundary, with changes in the switch trial parameter driving these improvements. To quantitatively compare the changes in drift rate and decision boundary, we performed a paired *t*-test on the difference of switch costs between Block 1 and 10, and found that decision boundary decreased significantly more than drift rate (*t*_(304)_ = 1378.1, *p* < 0.001, *d* = 80.704; Figure 6C).

**Figure 7:**
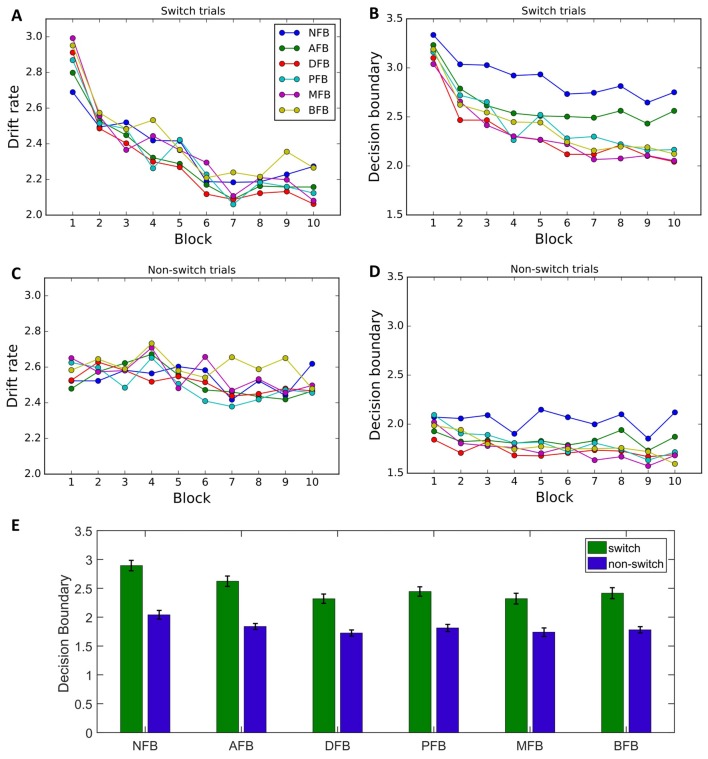
DDM data by condition.** (A–D)** Group level parameters for each feedback condition for switch trials and non-switch trials, drift rate, decision boundary. Results indicate that behavioral changes by condition are primarily due to differences in decision boundary. **(E)** Decision boundary by condition and trial type. Results indicate an overall decrease in decision boundary as feedback motivates good performance on switch trials, with the decrease being driven by the switch trial boundary. Error bars represent within-subject errors.

To determine what effect different feedback conditions had on decision making processes we looked at the effect of condition on the model parameters (Figures 7A–D). We found a main effect of Condition on decision boundary (*F*_(9,295)_ = 5.46, *p* < 0.001, $\eta_{\text{p}}^{2}$ = 0.084), but not on drift rate (*F*_(9,295)_ = 0.9, *p* = 0.484, $\eta_{\text{p}}^{2}$ = 0.015). Furthermore, the interaction between trial type and feedback was significant for decision boundary (Trial Type × Condition: *F*_(5,299)_ = 3.23 *p* = 0.007, $\eta_{\text{p}}^{2}$ = 0.708), but not drift rate (Trial Type × Condition: *F*_(5,299)_ = 0.42, *p* = 0.834). To investigate which conditions are driving the interaction we conducted *post hoc* two-tailed independent *t*-tests comparing the average switch cost across conditions and found that the NFB was not significantly different than AFB (*t*_(97)_ = 0.305, *p* = 0.7608), a trending difference from DFB (*t*_(99)_ = 1.604, *p* = 0.112) and BFB (*t*_(92)_ = 1.568, *p* = 0.12), and a significant difference from PFB (*t*_(96)_ = 1.79, *p* = 0.077, *d* = 0.362), and MFB (*t*_(95)_ = 1.98, *p* = 0.051, *d* = 0.402). These results suggest that feedback conditions that convey information in relation to switch performance are driving the Trial Type × Condition interaction (see Figure 7E). These results indicate that differences in switch cost for different feedback conditions also originated from differences in decision boundary. Finally, a non-significant 3-way interaction between Block, Condition and Trial Type for drift rate (*F*_(45,1475)_ = 1.1, *p* = 0.299) and decision boundary (*F*_(45,1475)_ = 1.25, *p* = 0.123) indicated that feedback did not affect changes in switch costs during training (Supplementary Figure S3).

### Training Transfer

**Figure 8:**
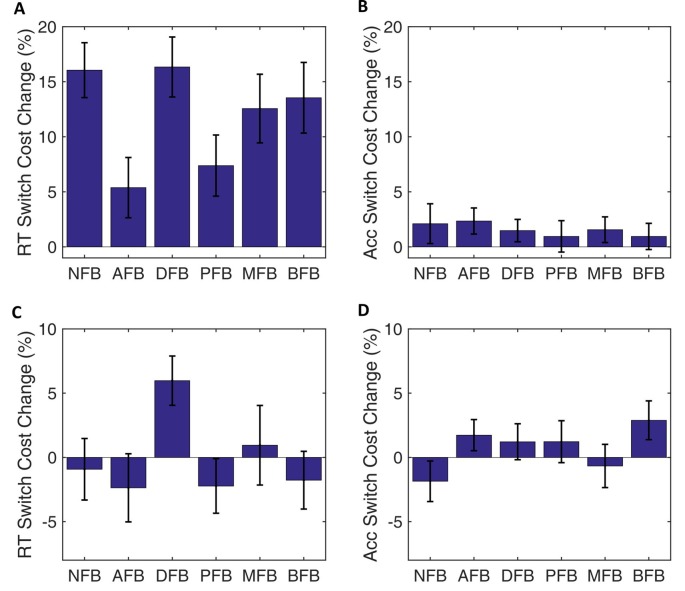
Transfer behavioral data. Change in switch cost from blocks pre- to post-test blocks for RT and Accuracy by condition. **(A,B)** Performance for the same task (i.e., green or blue, circle or square) as in training but with no feedback. **(C,D)** Performance during a novel task (i.e., standing or sitting, lion or tiger) with no feedback. Results indicate that training transferred to familiar but not novel task. Error bars represent standard errors.

To investigate transfer of training we looked at performance on pre- and post-training blocks with both familiar (blue and green circles and squares) and novel (standing and sitting lions and tigers) tasks. Paired *t*-tests on RT and accuracy between pre- and post-training blocks showed similar speed-accuracy trade-offs as found in training (see Supplementary Figure S4). However, the changes in switch costs did not transfer to novel tasks (RT: *t*_(304)_ = 0.03, *p* = 0.979, *d* = 0.002 and Accuracy: *t*_(304)_ = 1.29, *p* = 0.200, *d* = 0.101; Supplementary Figure S5). Finally, a one-way ANOVA failed to show a difference in novel tasks across condition in switch cost RT (*F*_(5,299)_ = 1.83, *p* = 0.109, $\eta_{\text{p}}^{2}$ = 0.030) or in switch cost Accuracy (*F*_(5,299)_ = 1.23, *p* = 0.29, $\eta_{\text{p}}^{2}$ = 0.020; Figure 8).

**Figure 9:**
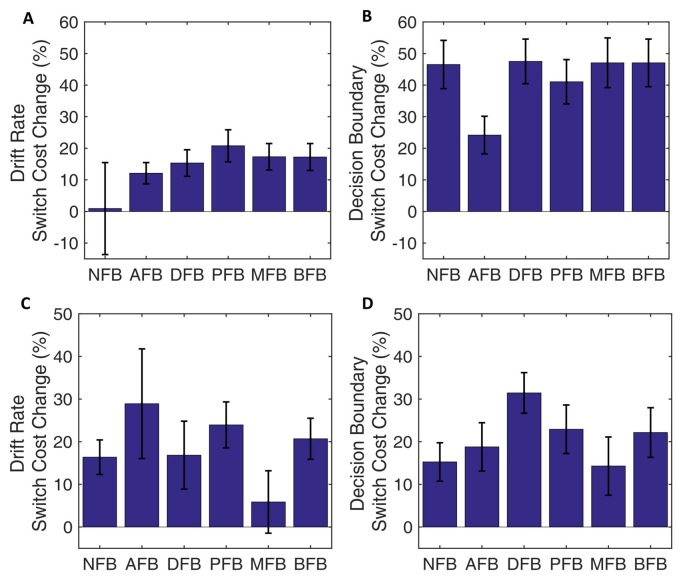
Transfer DDM data. Change in switch costs from pre- to post-test blocks for drift rate and decision boundary by condition. **(A,B)** Same task as in training blocks but with no feedback. **(C,D)** Novel task with no feedback. Results indicate that participants applied the same speed-accuracy trade-off as in training but there were no differences between conditions. Error bars represent standard errors.

Similar to drift diffusion parameter changes across training, drift rate and decision boundary *t*-tests showed a significant decreases between pre- and post-training blocks with a larger decrease in decision boundary (Supplementary Figure S6). Furthermore, there was a significant decrease in switch costs of both parameters for both familiar and novel tasks (drift rate: *t*_(304)_ = 5.20, *p* < 0.001, *d* = 0.394 and *t*_(304)_ = 5.86, *p* < 0.001, *d* = 0.457, respectively; decision boundary: *t*_(304)_ = 14.27, *p* < 0.001, *d* = 0.912 and *t*_(304)_ = 9.15, *p* < 0.001, *d* = 0.639, respectively; Supplementary Figure S7). This result indicates that the speed-accuracy trade-off change over training transferred to both familiar and novel tasks. However, a non-significant one way ANOVA on the switch cost difference from pre- to post-test indicates this speed-accuracy trade-off did not differ across conditions (drift rate: *F*_(5,299)_ = 1.01, *p* = 0.411; decision boundary: *F*_(5,299)_ = 1.29, *p* = 0.269; Figure 9).

## Discussion

In this study we investigated the effects of feedback and training on task-switching performance. Behavioral results showed that both task feedback and training had an effect on task switching performance as reflected by differences in switch costs across feedback conditions and across blocks. The behavioral data (RT and accuracy) indicated a change in strategy over the course of training, but the extent to which each condition drove differences in performance was unclear due to substantial variation in speed and accuracy within each condition. We used Drift Diffusion Modeling (DDM) to jointly account for both RT and accuracy allowing for explicit modeling of the speed-accuracy trade-off. The effects of training—reduction in RT switch costs—were found to be driven by the reduction in the decision boundary, while a simultaneous but smaller reduction in drift rate only served to partly counter such effects. DDM results revealed that differences in performance across feedback conditions were driven by differences in decision boundary, but not drift rate. In comparison to when no switch specific feedback was given, feedback that motivated faster performance on switch trials (e.g., Difficulty, Monetary, Punishment and Block FB conditions) led to a decreased decision boundary, reflecting speed-accuracy trade-offs. In sum, DDM showed that differences between conditions were underlied by differences in decision boundary, which was not evident from the behavioral data alone.

DDM parameter analysis revealed that participants accumulated information slower and used higher decision boundaries on switch compared to non-switch trials. These findings are in line with the interpretation that drift rates primarily reflect carry-over interference from the task on the previous non-switch trial while a larger decision boundary reflects a preparatory response to adapt to more difficult trials (Karayanidis et al., 2009; Schmitz and Voss, 2012). Moreover, the continuous decrease in drift rate and decision boundary was found only on switch trials while it stayed relatively constant on non-switch trials, reflecting that changes in performance over the course of training were due to changes in the decision process on switch trials. Learning that is reflected in the decrease of decision boundary is consistent with other training studies (Dutilh et al., 2011; Petrov et al., 2011; Liu and Watanabe, 2012; Zhang and Rowe, 2014). Such decreases have been interpreted as a change in behavior due to complying with speed-accuracy tradeoff instructions. Another possible interpretation of the decreased decision boundary is that it reflects task learning (Dutilh et al., 2011). Zhang and Rowe (2014) found that when an untrained stimulus was tested, decision boundary did not change while drift rate did, suggesting that the decision boundary reflected learning that transferred across tasks.

The decrease in drift rate over the course of training is more difficult to explain in terms of learning. Learning, as studied outside of task-switching research, has typically been shown to be driven by an increase in drift rate rather than a decrease (Dutilh et al., 2011; Petrov et al., 2011; Liu and Watanabe, 2012; Zhang and Rowe, 2014). Thus, one possible explanation for the decrease in drift rate could be fatigue that arises over the course of the task (Schmiedek et al., 2007). However, the largest decrease occurs within the first few blocks with incremental changes thereafter and only on switch trials suggesting that this effect may reflect more meaningful changes in the decision process itself.

In our study, the decrease in decision boundary on switch trials may reflect learning to anticipate when switches would occur and participants choosing increased speed at the expense of accuracy. This learning effect is in line with previous research showing that task switching performance is altered by task predictability (Monsell et al., 2003; Vandierendonck et al., 2010). For example, Monsell et al. (2003) found that participants returned to baseline RT just one trial after a predictable switch compared whereas it took several trials after a unpredictable switch. This result suggests that participants’ expectations about the switch influence switching performance. Previous research has also shown that predictability can influence transfer of task-switching training. For example, Minear and Shah (2008) found that groups trained with unpredictable task switching, but not predictable switching, transferred to an untrained switch task. Our behavioral results, indicating a lack of transfer, are in line with this finding but our DDM analysis suggests that participants are applying the same speed-accuracy trade-off that was learned over the course of training.

Adjusting speed-accuracy trade-off over the course of training also explains why some feedback conditions had an overall decrease in decision boundaries on switch trials. An effect of task learning is evident in the Accuracy and NFB conditions where feedback did not motivate optimizing the speed-accuracy trade-off on switch trials compared to non-switch trials. In comparison, the Difficulty, Punishment, Monetary and BFB conditions, switch trial performance was rewarded more for correct and faster performance leading to an overall decrease in switch trial decision boundary which explains the overall decrease in RT for these conditions.

Finally, our results are relevant to the task switch training literature in that feedback can be used to successfully motivate behavior that coincides with training goals. To achieve training goals, behavior must change on the relevant task dimension. In the case of task switching training the typical goal is to improve the ability to switch to another task. While results in the present study indicate that feedback is not improving task switching ability, we show that feedback can motivate participants to specifically modify behavior on switch trials. This result indicates that reward structures, if properly constructed to align with training goals, may be able to modify behavior in a manner consistent and beneficial to training outcomes.

## Conclusion

We found that both feedback and training can have significant effects on task-switching performance. We used DDM modeling to account for speed-accuracy trade-offs and, for the first time, to show how decision processes change over the course of task-switching training. Specifically, we found that participants show a decreased drift rate and increased decision boundary on switch trials compared to non-switch trials, possibly reflecting task set interference and a preparatory response before more difficult trials. Moreover, the change in switch cost over the course of training was driven by a decrease in the decision boundary, reflecting speed-accuracy trade-offs. Finally, task feedback effects on RT switch cost were also driven by differences in decision boundary, but not drift rate. These results help show that learning is not necessarily best described as improvements of task performance, but instead should be characterized by how participants adapt their behavior to the training procedure that are made most relevant to them by feedback on their performance. Overall, our results suggest that DDM can provide additional insight into feedback and training effects on task-switching performance.

## Author Contributions

RCH and PK made equal contributions and are co-first authors. ARS and RCH designed the study. RCH collected the data. RCH, PK and SR analyzed the data. RCH, PK, SR, PS and ARS contributed to the writing of the manuscript.

## Conflict of Interest Statement

The authors declare that the research was conducted in the absence of any commercial or financial relationships that could be construed as a potential conflict of interest.
