# Supplementary material for: The Influence of Feedback on Task-Switching Performance: A Drift Diffusion Modeling Account
Source: Front Integr Neurosci. 2018 Feb 2;12:1. doi: 10.3389/fnint.2018.00001 (PMC5801306; doi:10.3389/fnint.2018.00001)
Supplement: Supplementary file 1 [file Data_Sheet_1.pdf]

Supplementary Material

The Influence of Feedback on Task-Switching Performance: A Drift Diffusion Modeling Account

Russell Cohen Hoffing*, Povilas Karvelis*, Sam Rupprechter, Peggy Seriès, Aaron R Seitz
***Co-first author.**
Correspondence: Aaron R Seitz, aseitz@ucr.edu

1 Supplementary Figures and Tables

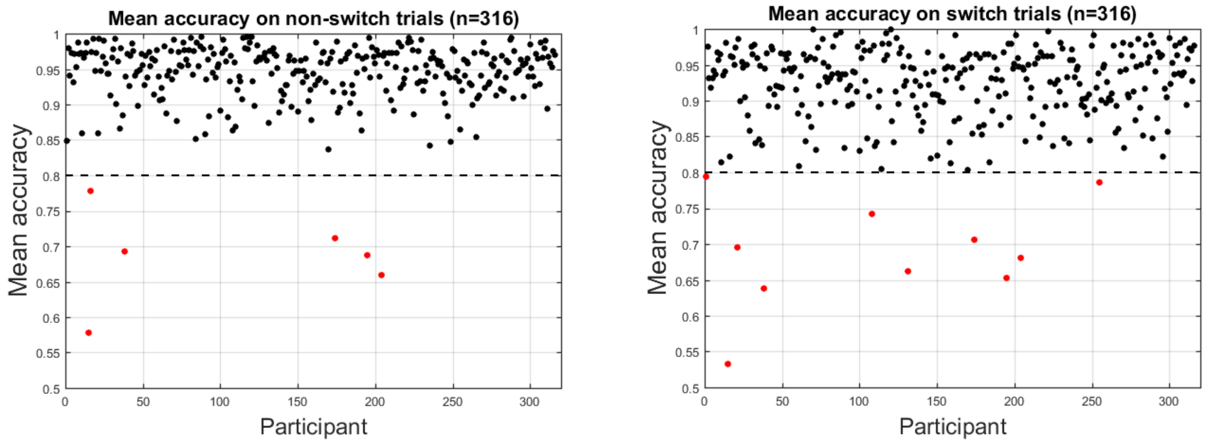

Figure S1. Mean accuracy for each individual on switch (right panel) and non-switch (left panel) trials. The dashed line indicates exclusion criterion and red dots are individuals that did not satisfy the criterion. Across the two trial types this resulted in excluding 11 participants.

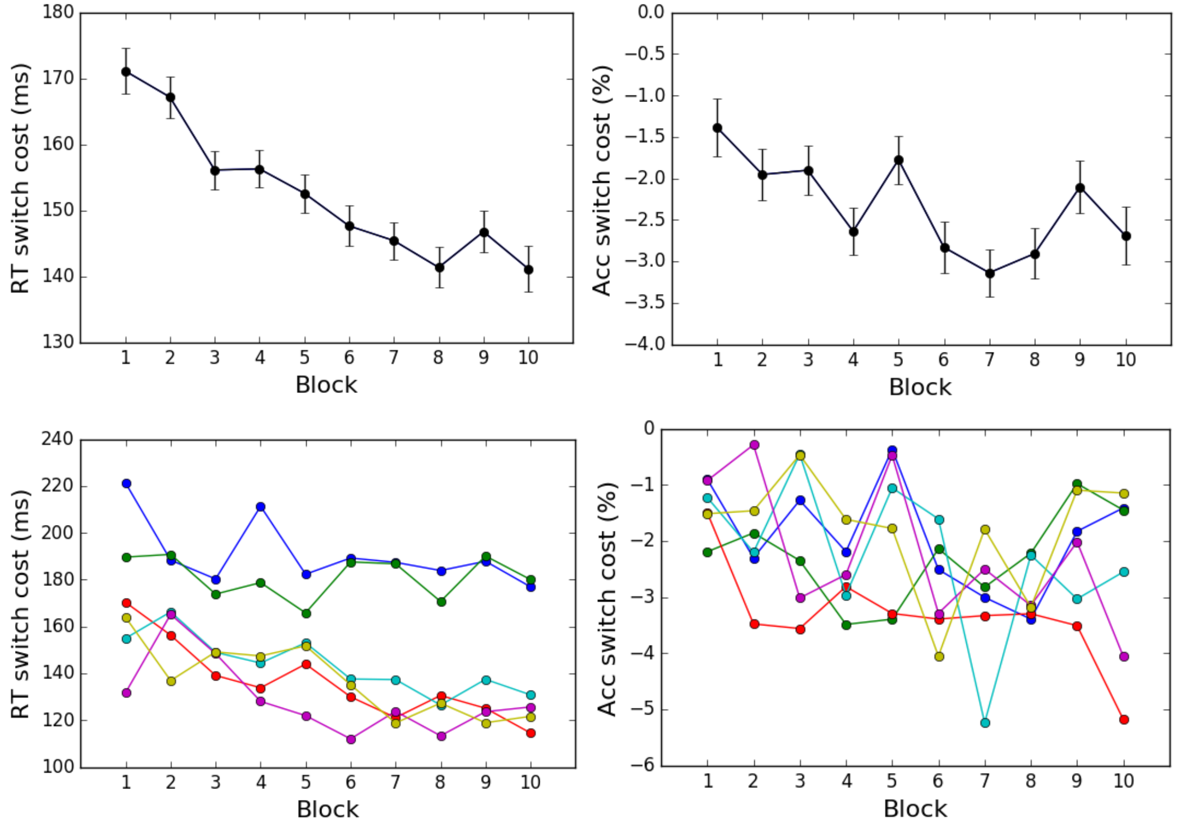

Figure S2. Switch costs calculated by taking the difference between switch and non-switch trials for Reaction Time (left column) and Accuracy (right column), averaged across conditions (top row) and averaged within each condition (bottom row). The results are qualitatively identical to the switch costs calculated as the ratio between switch and non-switch trials as presented in the main text.

**Model**	**DIC**
**DDM_1 (*v,* and *a* depend on *trial type* and *block; t* fixed; *z=0.5*)**	**31088**
DDM_2 (*v* depends on *trial type* and *block; a, t* fixed; *z=0.5*)	56681
DDM_3 (*a* depends on *trial type* and *block; v, t* fixed; *z=0.5*)	40582
DDM_4 (*v,* and *a* depend on *trial type* only*; t* fixed; *z=0.5*)	68488
DDM_5 (*v* depends on *trial type* only*; a, t* fixed; *z=0.5*)	77260
DDM_6 (*a* depends on *trial type* only*; v, t* fixed; *z=0.5*)	71611

Table S1. Model evidence for different model assumptions. Lower DIC value indicates a better model. The best model here is DDM_1.

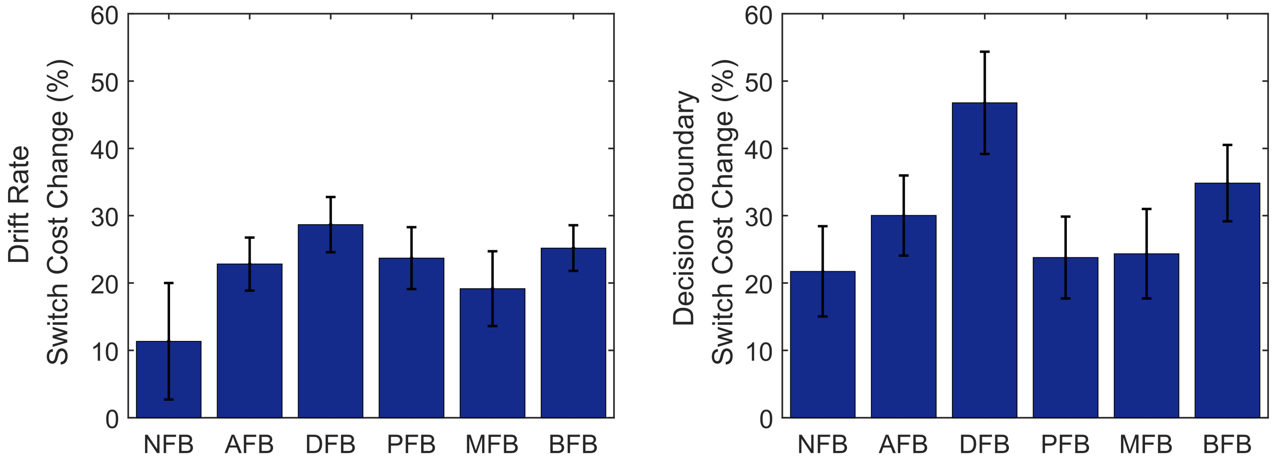

Figure S3: Change in switch cost drift rate and decision boundary by condition from block 1 to 10. A one-way ANOVA on the switch cost difference between Block 1 and 10 showed no significant difference between conditions for Drift Rate ($F(5,299)=1.28$, $p=0.27$) and a trend of difference in Decision Boundary ($F(5,299)=1.79$, $p=0.12$).

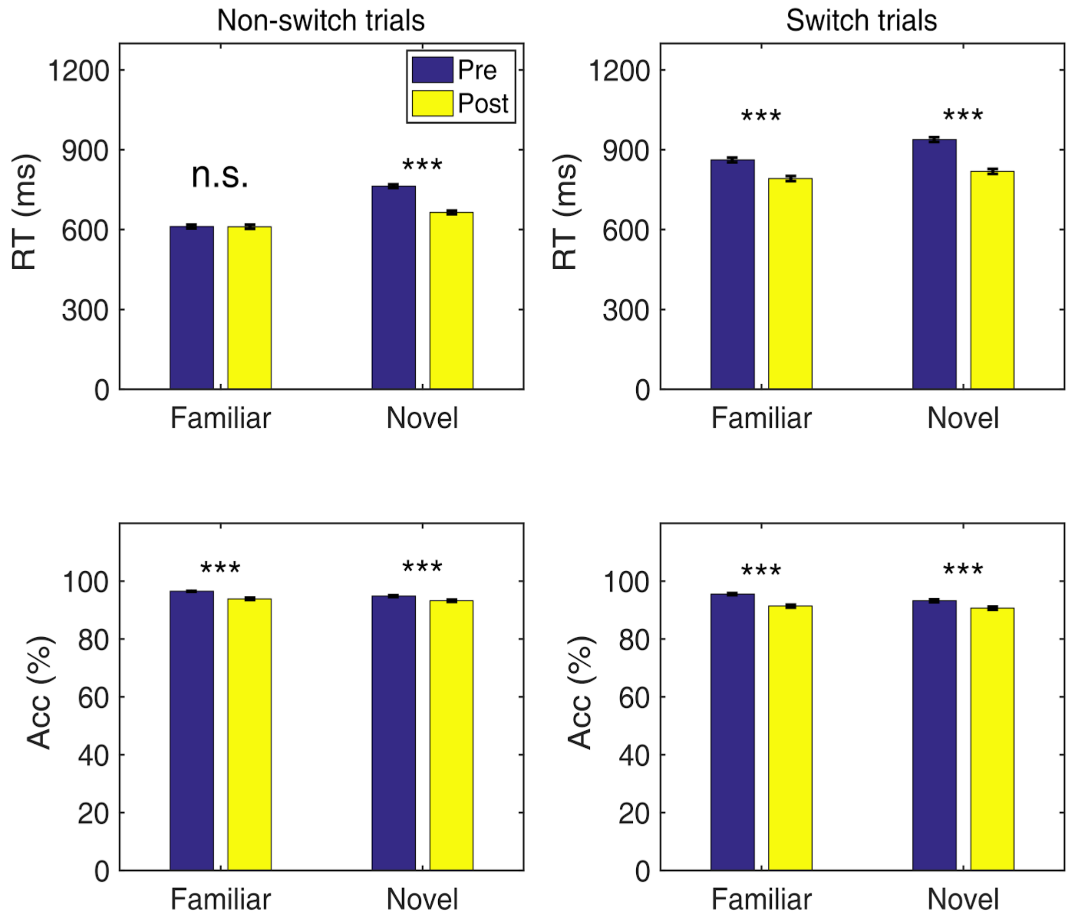

Figure S4. RT and Accuracy for pre- and post-training blocks for familiar and novel tasks and for switch and non-switch trials. RT of non-switch trials, familiar: t(304) = 0.08, p = 0.934, d = 0.007; novel: t(304) = 18.27, p < 0.001, d = 0.828. RT of switch trials, familiar (t(304) = 8.32, p < 0.001, d = 0.440), novel: t(304) = 14.42, p < 0.001, d = 0.748; Accuracy of non-switch trials, familiar: t(304) = 6.52, p < 0.001, d = 0.528; novel: t(304) = 3.40, p < 0.001, d = 0.253. Accuracy of switch trials, familiar: t(304) = 7.67, p < 0.001, d = 0.552; novel: t(304) = 3.87, p < 0.001, d = 0.295. *** - p < 0.001, n.s. - p > 0.05.

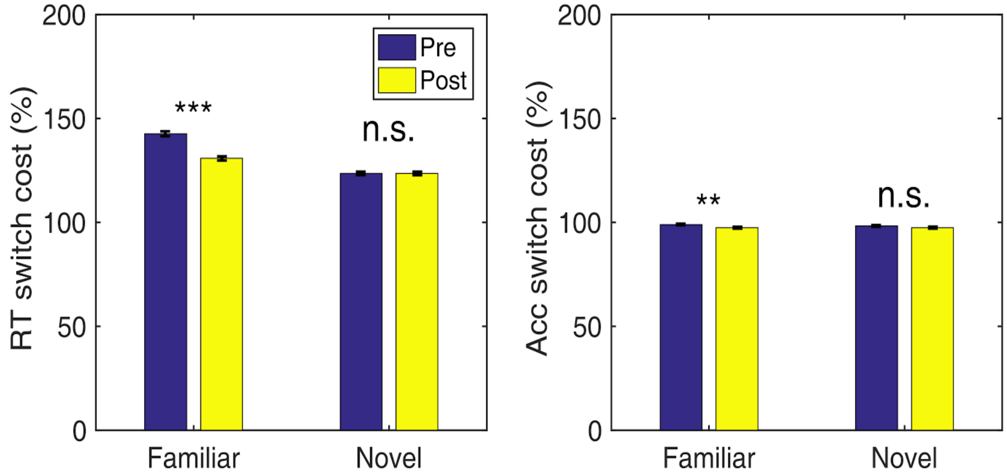

Figure S5. RT and Accuracy switch cost on pre- and post-training blocks for familiar and novel tasks. RT switch cost, familiar tasks: t(304) = 9.98, p < 0.001, d = 0.610; novel tasks: t(304) = 0.03, p = 0.979, d = 0.002. Accuracy for switch cost, familiar tasks: t(304) = 2.97, p = 0.003, d = 0.228; novel tasks: t(304) = 1.29, p = 0.200, d = 0.101. *** - p < 0.001, ** - p < 0.01, n.s. - p > 0.05.

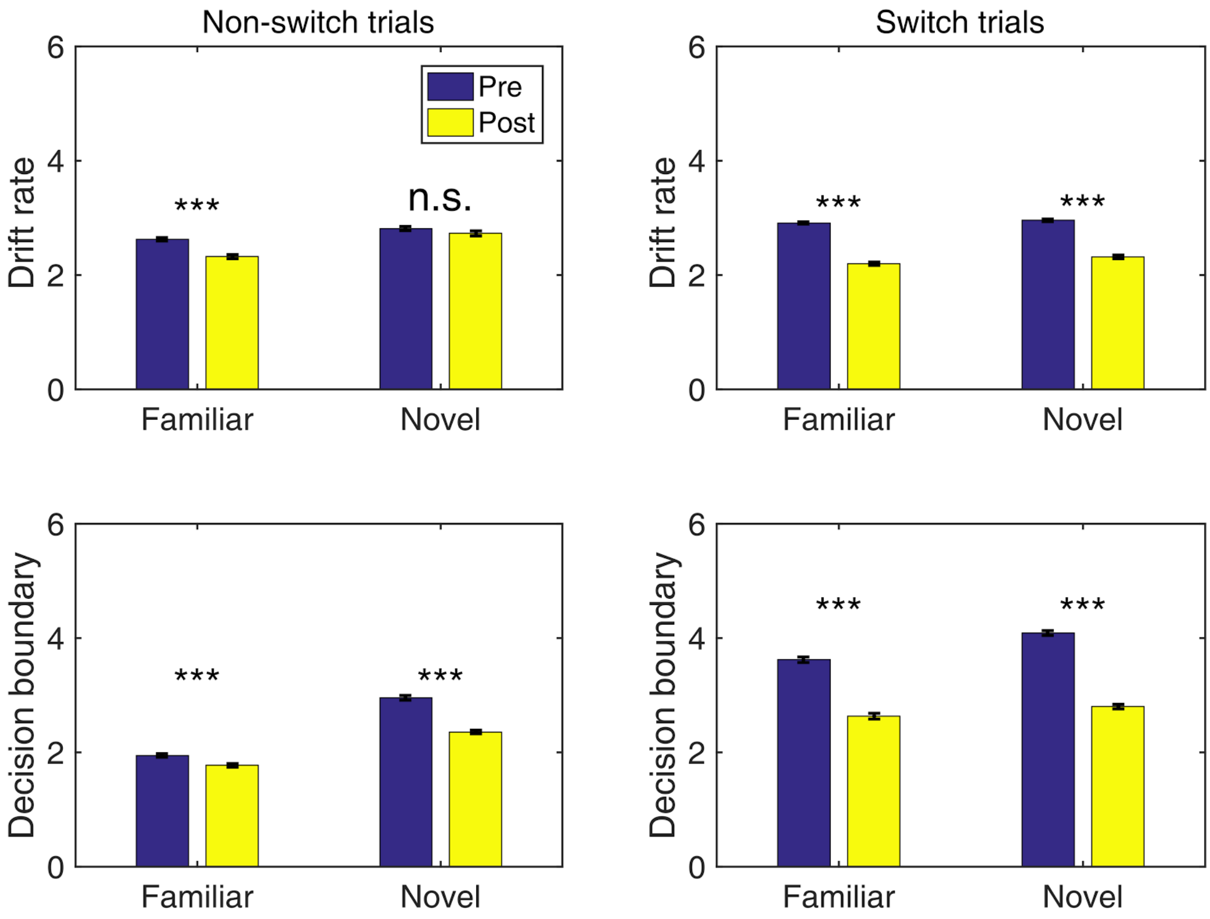

Figure S6. Drift rate and decision boundary on pre- and post-training blocks for familiar and novel stimuli for switch and non-switch trials. Drift rate on non-switch trials, familiar: t(304) = 6.36, $p < 0.001$, d = 0.514; novel: t(304) = 1.76, p = 0.079, d = 0.120. Drift rate on switch trials, familiar: t(304) = 21.97, $p < 0.001$, d = 1.604; novel: t(304) = 18.29, $p < 0.001$, d = 1.320; Accuracy on non-switch trials, familiar: t(304) = 4.00, $p < 0.001$, d = 0.324; novel: t(304) = 14.51, $p < 0.001$, d = 0.907. Accuracy on switch trials, familiar: t(304) = 19.61, $p < 0.001$, d = 1.129; novel: t(304) = 31.26, $p < 0.001$, d = 1.770. *** - $p < 0.001$, n.s. - $p > 0.05$.

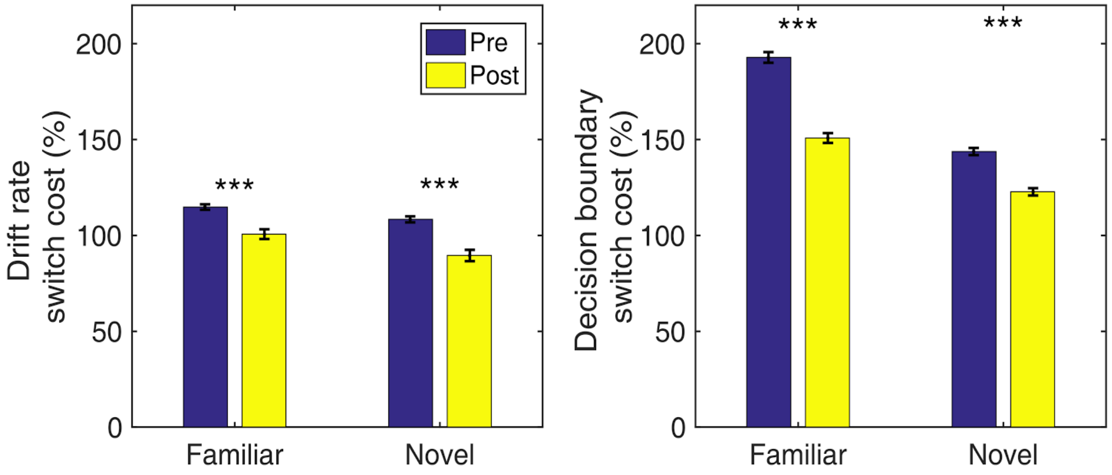

Figure S7. Drift rate and decision boundary switch cost on pre- and post-training blocks for familiar and novel stimuli. Drift rate for switch cost, familiar tasks: $t(304) = 5.20$, $p < 0.001$, $d = 0.394$; novel tasks: $t(304) = 5.86$, $p < 0.001$, $d = 0.457$. Decision boundary for switch cost, familiar tasks: $t(304) = 14.27$, $p < 0.001$, $d = 0.912$; novel tasks: $t(304) = 9.15$, $p < 0.001$, $d = 0.639$. *** - $p < 0.001$.
